# Supplementary material for: Exploring leprosy perceptions in South Sulawesi, Indonesia: A mixed-methods study on knowledge, attitudes, practices, and stigma
Source: PLOS Glob Public Health. 2025 Dec 26;5(12):e0005675. doi: 10.1371/journal.pgph.0005675 (PMC12742724; doi:10.1371/journal.pgph.0005675)
Supplement: S1 Text — (DOCX) [file pgph.0005675.s001.docx]

**S1_Text. The detailed description of the model explaining the relationship between the dependent and independent variables using a Bayesian approach**

Let $\left\{ X_{1},X_{2},\cdots,X_{J} \right\}$ be the set of all variables under study, and for variable $X_{j}$ ($j=1,2,\cdots,J$) let $\left\{ X_{j1},X_{j2},\cdots,X_{jK(j)} \right\}$ be the set of its categories, where $K\left( j \right)$ and $J$ are the number of the categories of variable $X_{j}$ and the number of variables under study, respectively. Let also $Y_{i;S}$ be the score of the measure $S\in\left\{ KAP, EMIC-CSS, SDS \right\}$ for the $i^{\mathrm{th}}$ participant, and $X_{jk;i}\in\left\{ 0,1 \right\}$ is the $k$^th^ component of the non-reference categorical vector for the $i^{\mathrm{th}}$ participant

$$\left( X_{j1;i},X_{j2;i},\cdots,X_{jK\left( j \right);i} \right)$$

where for $k=1,2,\cdots,K\left( j \right)$

$$X_{jk;i}=\left\{ \begin{matrix} 1; & if the i^{\mathrm{th}} participant has category X_{jk} of variable X_{j} \\ 0; & \mathrm{otherwise} \end{matrix} \right.$$

Our model is

$$Y_{i;S}\sim\mathrm{Normal}\left( \mu_{i;S},\tau_{i;S} \right)$$

$$\mu_{i;S}=b_{0;S}+\sum_{j=1}^{J} \sum_{k=1}^{K\left( j \right)} b_{jk;S}X_{jk;i}$$

The parameters of the model were estimated using a Bayesian approach. The prior distribution of the intercept $b_{0;S}$, the slope $b_{jk;S}$, and the precision $\tau_{i;S}$ are is uniform $(0,q)$ where $q$ is the maximum score that probably can be achieved by a participant, $\mathrm{Normal}(0, 0.001)$, and $\mathrm{gamma}(1.5, 0.01)$ for each for each $i$, $j$, $k$, and $S$.

With the reference category, there are $K\left( j \right)+1$ categories for each variable $X_{j}$. So, the complete categorical vector for the $i^{\mathrm{th}}$ participant is

$$\left( X_{j0;i},X_{j1;i},X_{j2;i},\cdots,X_{jK\left( j \right);i} \right)$$

with the value of a component is 1 and 0 for the others. For instance, for the variable sex with female as the reference, female participants can be expressed as $\left( 1,0 \right)$, and $\left( 0,1 \right)$ for male participants. Another example, for the variable education with the categories no-education (the reference), primary education, secondary education, and university education, the expression for the categories are $\left( 1,0,0,0 \right)$, $\left( 0,1,0,0 \right)$, $\left( 0,0,1,0 \right)$, and $\left( 0,0,0,1 \right)$, respectively. The list of the variables and the corresponding categories are as in the Table I. The analyses were performed in R version 4.3.3 and RStudio version 2024.04.0. For estimating the model parameters, we used WinBUGS version 1.4.3 from the https://www.mrc-bsu.cam.ac.uk page.

Table A. The list of the variables and the corresponding categories that were involved in the model

| **No (**$\boldsymbol{j}$**)** | **Variable (**$\boldsymbol{X}_{\boldsymbol{j}}$**)** | **Number of Categories (**$\boldsymbol{K}\left( \boldsymbol{j} \right)$**)** | **Category (**$\boldsymbol{X}_{\boldsymbol{jk}}$**)** | |
| --- | --- | --- | --- | --- |
| 1 | Age | 3 | 1 | Age < 30 |
|  |  |  | 2 | Age 30-49 |
|  |  |  | 3 | Age >= 50 |
| 2 | Marital Status | 3 | 1 | Married/Living Together |
|  |  |  | 2 | Separated |
|  |  |  | 3 | Never Married |
| 3 | Education | 4 | 1 | No Education |
|  |  |  | 2 | Primary |
|  |  |  | 3 | Middle/Secondary |
|  |  |  | 4 | University |
| 4 | Occupation | 5 | 1 | Health Care Worker |
|  |  |  | 2 | Self-Employee |
|  |  |  | 3 | Farmer |
|  |  |  | 4 | Working in the Household |
|  |  |  | 5 | Temporary Work/Unemployed |
| 5 | Gender | 2 | 1 | Female |
|  |  |  | 2 | Male |
| 6 | Relationship with person affected by leprosy (PAL) | 2 | 1 | Have no Relationship with PAL |
|  |  |  | 2 | Have Relationship with PAL |
| 7 | Monthly household income (USD) | 4 | 1 | Income < 95 |
|  |  |  | 2 | Income 95-189 |
|  |  |  | 3 | Income >= 190 |
|  |  |  | 4 | Don't know/No Answer |
| 8 | Area of residence | 2 | 1 | Non-Urban |
|  |  |  | 2 | Urban |
