# Supplementary material for: Exploring leprosy perceptions in South Sulawesi, Indonesia: A mixed-methods study on knowledge, attitudes, practices, and stigma
Source: PLOS Glob Public Health. 2025 Dec 26;5(12):e0005675. doi: 10.1371/journal.pgph.0005675 (PMC12742724; doi:10.1371/journal.pgph.0005675)
Supplement: Attachment — Submitted filename: Comment.docx [file pgph.0005675.s002.docx]

Comment:

1. In your introduction you expressed the opinion that the stagnation in case detection may be related to stigma and discrimination. I agree with you, but as the infection progresses in a person, delays in early case detection are usually followed by a high rate of discovery of patients with advanced levels of disability. Is this also the case in your research area? If this is the case, this data needs to be mentioned to increase the urgency of carrying out this research
2. As we know, in principle, the mix method approach in research is based on certain objectives. The choice of mix method was based on the consideration that with this approach results would be obtained to answer the objectives better when compared to using one of them. It seems that you need to provide an explanation in deciding to choose this approach, along with an explanation of the design, defining sample / respondents and stages of conducting the research.
3. Population determination and sampling procedures need to be explained in more detail. How do you determine the population and sample / respondents for each research stage, what connecting procedures and analysis techniques used?
4. In lines 612 - 613 you state the weaknesses of the cross-sectional design of this research, but actually what you are doing is a mixed method approach which has the strength to achieve the research objectives. What do you think about this?
5. Because you involve program staff in the study, it is best that at the conclusion you need to provide suggestions in more detail for improving the program
