# Supplementary material for: Exploring leprosy perceptions in South Sulawesi, Indonesia: A mixed-methods study on knowledge, attitudes, practices, and stigma
Source: PLOS Glob Public Health. 2025 Dec 26;5(12):e0005675. doi: 10.1371/journal.pgph.0005675 (PMC12742724; doi:10.1371/journal.pgph.0005675)
Supplement: Attachment — Submitted filename: Response to reviewer_20082025.docx [file pgph.0005675.s003.docx]

**Exploring leprosy perceptions in South Sulawesi, Indonesia: a mixed-methods study on knowledge, attitudes, practices, and stigma**

We sincerely thank the reviewers for their valuable comments and constructive suggestions, which have greatly improved the manuscript. Below, we provide our detailed responses to each point, together with the corresponding revisions in the manuscript.

**Reviewer 1**

This is a well-constructed study, and the data is very clearly presented. I think it is important in showing that leprosy stigma and lack of knowledge, particularly about the disease in its early symptoms, continue to have a significant impact on people’s lives in Sulawesi, despite Indonesia’s status as having “eliminated” the disease. I liked that the authors suggest some of the complex reasons why people might have certain beliefs. I agree that the long incubation period is one of the reasons people in many parts of the world speculate on ways they might have been exposed, often leading to misconceptions.

In terms of the belief that leprosy is hereditary, it is also easy to see why people might think this, as leprosy susceptibility might be inherited – you could point out that household contacts (often family members) are at a higher risk, and this, coupled with possible susceptibility running in families, could make it seems like leprosy itself could be passed down from parent to child. The belief that one of your participants discussed of the possibility of leprosy transmission through livestock feces is one that is the subject of a few ongoing studies currently.

Question 1. In designing demographic questions, I think it is important for researchers to be aware of gender diversity rather than sticking to the gender binary (male/female) to have participants self-identity. This is perhaps even more significant since the Bugis-Makassar are known (at least in the contemporary anthropology literature) as recognizing five gender identities, and with these different identities, come different roles and expectations that also might play a part in the experience of having leprosy. I’m not sure if you have this data on whether people identified with gender/sex assigned at birth or not, but if not, this could be something to consider for future studies. Sharyn Graham-Davies has written extensively on this, but this is her book that focuses on the Bugis culture.

Davies, Sharyn Graham. “Challenging Gender Norms: Five Genders Among Bugis in Indonesia (Case Studies in Cultural Anthropology) by Grahan Davies, Sharyn (2006) Paperback. I know this text might not be readily accessible, but this summarizes some of her research.

<https://www.bbc.com/travel/article/20210411-asias-isle-of-five-separate-genders>

**Reply:** Thank you for this valuable comment. In the present study, we did not collect data on participants’ self-identification regarding gender. We have noted this as a limitation of our survey tool and proposed that future work incorporate context-specific considerations (e.g., the use of rapid ethnographic tools to assess such aspects) prior to finalizing survey instruments (page 24, line 11-6).

Question 2. This is just a suggestion, but I think it can humanize participants you are quoting to use pseudonyms rather than numbers. If you choose to use pseudonyms, you can use typical local name for the age of the participants.

**Reply:** We agree with the reviewer’s suggestion to use pseudonyms rather than study numbers to humanize the interviewees. We have revised the manuscript accordingly and added a sentence in the Methods section to clarify this change: “Pseudonyms were used for each interviewee to safeguard confidentiality and preserve a more personal narrative.” (page 7, line 128-9).

I just have a few more minor notes by line of the manuscript:

| **Suggestions from reviewer 1** | **Responses from authors** |
| --- | --- |
| Line 77-78: “The impact of leprosy and the associated disabilities has taken a multifaceted toll on the physical, psychological, social, and economic aspects of those affected.” Suggested rewording: “Leprosy and the associated disabilities often have a negative impact on the physical, psychological, social, and economic aspects of the lives of people affected.” | We have revised the sentence as suggested (page 4, line 15-6). |
| Line 99: “from Bugis-Makassar ethic” – maybe “from Bugis-Makassar ethnic groups” | We have revised the term as suggested (page 5, line 9). |
| Line 244: You might include the different word origins. You mention kandala as a Bugis-Makassar specific term, but I think kusta comes from Sanskrit and Hindu influence in Indonesia. | We have added the suggested explanation about the origin of kusta term (page 10, line 22-23). |

**Reviewer 2**

This article is interesting because leprosy is still a health problem in several countries. Public knowledge, stigma and discrimination seem to be urgent matters to be addressed in government programs. Research findings to support program development need to be carried out and presented as a reference. If you agree, I have attached some suggestions regarding this article.

| **Suggestions from reviewer 2** | **Responses from authors** |
| --- | --- |
| 1. In your introduction you expressed the opinion that the stagnation in case detection may be related to stigma and discrimination. I agree with you, but as the infection progresses in a person, delays in early case detection are usually followed by a high rate of discovery of patients with advanced levels of disability. Is this also the case in your research area? If this is the case, this data needs to be mentioned to increase the urgency of carrying out this research | Thank you for this important comment. We agree that delays in case detection typically result in higher proportions of disability at diagnosis. Unfortunately, we did not have access to district-level data on disability grading for our study area. However, national data indicate that approximately 6% of newly detected cases present with Grade 2 disability, underscoring the urgency of addressing stigma and strengthening early case detection. We have added this information to the introduction (page 4, line 6-7). |
| 2. As we know, in principle, the mix method approach in research is based on certain objectives. The choice of mix method was based on the consideration that with this approach results would be obtained to answer the objectives better when compared to using one of them. It seems that you need to provide an explanation in deciding to choose this approach, along with an explanation of the design, defining sample / respondents and stages of conducting the research. | Thank you for this suggestion. We have clarified the methodology in more detail as requested by the reviewer (page 4, line 27 up to page 5, line 4).  “The quantitative component, consisting of interview-administered questionnaires, provided measurable evidence on knowledge, attitudes and behaviour related to leprosy. The qualitative component, through semi-structured interviews (SSIs) and focus group discussions (FGDs), offered deeper insights into people’s experiences, perceptions and coping strategies. Integrating these findings allowed us to develop a richer and more nuanced understanding of leprosy-related stigma (Figure 1).” |
| 3. Population determination and sampling procedures need to be explained in more detail. How do you determine the population and sample / respondents for each research stage, what connecting procedures and analysis techniques used? | We have tried to clarify the methodology section in more detail as suggested by the reviewer.  The population and sampling procedures for both quantitative and qualitative components are described in the section “Study population and sampling”. For the quantitative approach this is page 5, line 16 up to page 6, line 4; and for the qualitative approach page 6, line 6-12.  We added an explanation regarding the connection between the quantitative and qualitative data collection:  Page 6, line 6-8: Survey participants who gave more detailed or insightful responses were invited to participate in the SSIs or FGDs.  Page 7, line 19-20: We conducted a preliminary analysis of the survey data prior to the SSIs and FGDs to help refine and identify targeted topics for deeper exploration. |
| 4. In lines 612 - 613 you state the weaknesses of the cross-sectional design of this research, but actually what you are doing is a mixed method approach which has the strength to achieve the research objectives. What do you think about this? | We agree with the reviewer and have removed this limitation from the manuscript and instead included the limitation suggested by Reviewer 1 about gender identity in Bugis-Makassar culture (page 24, line 11-6). |
| 5. Because you involve program staff in the study, it is best that at the conclusion you need to provide suggestions in more detail for improving the program | We have added specific suggestions related to HCWs (page 24, line 28-31). |
